# Inactivation of the complement anaphylatoxin C5a by secreted products of parasitic nematodes

**DOI:** 10.1016/j.ijpara.2009.10.006

**Published:** 2010-04

**Authors:** Dominic Rees-Roberts, Lisa M. Mullen, Kleoniki Gounaris, Murray E. Selkirk

**Affiliations:** Division of Cell & Molecular Biology, Department of Life Sciences, Imperial College London, London SW7 2AZ, UK

**Keywords:** Complement, C5a, Anaphylatoxin, Inflammation, Nematode, *Brugia malayi*, *Trichinella spiralis*

## Abstract

Given the importance of the complement anaphylatoxins in cellular recruitment during infection, the ability of secreted products from larval stages of *Brugia malayi* and *Trichinella spiralis* to influence C5a-mediated chemotaxis of human peripheral blood granulocytes in vitro was examined. Secreted products from *B. malayi* microfilariae almost completely abolished chemotaxis. This inhibition was blocked by phenylmethylsulphonyl fluoride, indicating the presence of a serine protease, which was subsequently shown to cleave C5a. In contrast, secreted products from *T. spiralis* infective larvae showed modest inhibition of C5a-mediated granulocyte chemotaxis, and this was blocked by potato carboxypeptidase inhibitor, an inhibitor of several metallocarboxypeptidases. Adult and larval stages of both parasites were demonstrated to secrete carboxypeptidases which cleaved hippuryl-l-lysine and hippuryl-l-arginine, and the *T. spiralis* enzyme was partially characterised. The data are discussed with reference to inflammation in parasitic nematode infection.

## Graphical abstract

**Figure:**
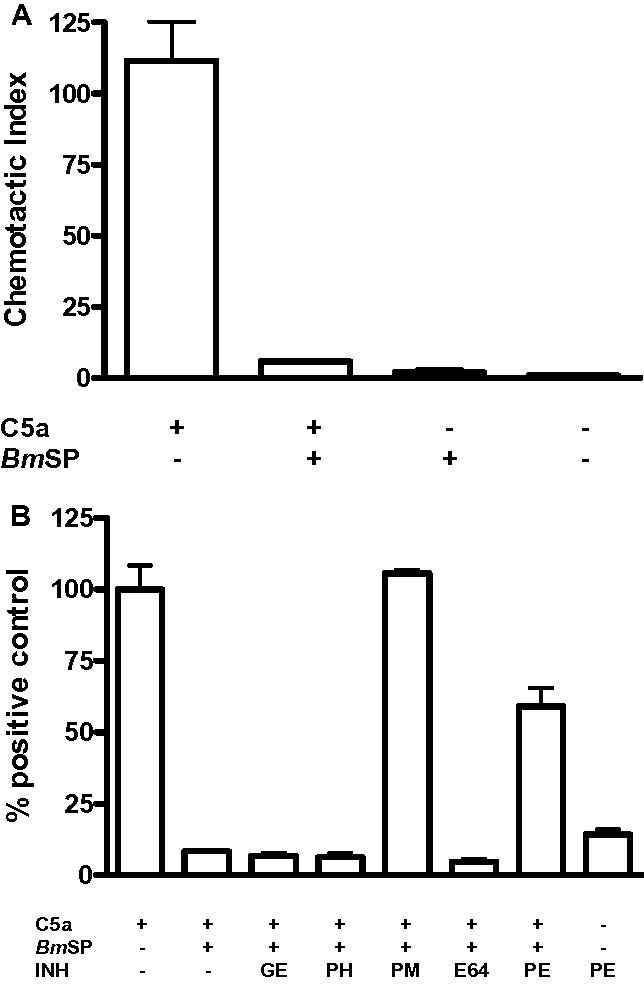

## 1 Introduction

The molecular basis for resistance of nematode parasites to host immunity is of interest given their persistence and inability to vary their antigenic profile. Inflammation does not always match that expected from secretion of potently antigenic material, for example in lymphatic filariasis, in which individuals frequently harbour high burdens of larval parasites in the circulatory system with no outward sign of infection (Maizels et al., 1995). In contrast, chemotherapy of heavily infected individuals invariably induces inflammatory responses as a result of trapping and killing of microfilariae, which may also occur following immunological clearance of parasites, suggesting that active infection suppresses inflammation (Maizels et al., 1995).

Modulation of cytokine networks by nematodes, and helminths in general, clearly influences inflammation and this is being exhaustively studied in a wide range of experimental systems. In contrast, biochemical and molecular studies of parasite anti-inflammatories has been limited (Maizels and Yazdanbakhsh, 2008). A number of tapeworm proteins inhibit neutrophil chemotaxis (Leid et al., 1987; Shepherd et al., 1991) and several nematode secreted enzymes mediate degradation of pro-inflammatory chemokines such as eotaxin and platelet-activating factor (Grigg et al., 1996; Culley et al., 2000). *Ancylostoma caninum* also secretes Neutrophil Inhibitory Factor (NIF), which binds the β integrin CD18/CD11b and inhibits neutrophil adhesion to vascular endothelial cells and subsequent activation (Moyle et al., 1994).

Complement plays multiple roles in both innate and adaptive immunity (Volanakis, 2002). Numerous early in vitro studies implicated complement as a factor in mediating adherence of myeloid cells to nematode parasites and subsequent killing, although this was generally restricted to larval stages with considerable differences in susceptibility between parasite species (Maizels et al., 1982). Opsonisation is known to be effected by the complement components C3b and C3bi, whereas cellular recruitment is directed primarily by the anaphylatoxins C3a and C5a (Volanakis, 2002). Nevertheless, the significance of these biological roles and relative contribution to anti-nematode immunity are not properly understood, due to a paucity of in vivo studies. Recently however, mice deficient in individual components of complement have been used to assess its importance in immunity to *Nippostrongylus brasiliensis*, a parasite which has an initial phase of migration through the skin to the lungs, followed by establishment and reproduction in the gut. Both factor B-deficient mice (defective in the alternative pathway) and C3-deficient animals (refractory to complement activation in general) had higher numbers of larvae in the lungs during primary infection, indicating a role for complement in parasite attrition during migration through somatic tissues (Giacomin et al., 2008b). Recruitment of eosinophils and aggregation of larvae in an air pouch model were reduced in these animals. Moreover, administration of the C5a receptor antagonist PMX53 also reduced recruitment of both eosinophils and neutrophils to the skin during the early phase of infection, indicating an important role for anaphylatoxins in this process (Giacomin et al., 2008b).

In this study we examined the ability of secreted products from larvae of two species of parasitic nematode to influence C5a-mediated chemotaxis of granulocytes in vitro. Secreted products of *Trichinella spiralis* muscle stage larvae showed a mild inhibition of the process due to a carboxypeptidase activity which was subsequently characterised. In contrast, secreted products of *Brugia malayi* microfilariae showed much more potent inactivation of chemotaxis, due to cleavage of C5a by a serine protease.

## 2 Materials and methods

### 2.1 Parasites

*Brugia malayi* adults and microfilariae were isolated from infected jirds purchased from TRS Laboratories (Athens, GA, USA) and cultured in serum-free RPMI 1640 for up to 5 days as previously described (Thomas et al., 1997). Adults and infective larvae of *T. spiralis* were recovered from outbred rats and cultured in serum-free RPMI 1640 for up to 3 days as previously described (Arden et al., 1997). Culture media were cleared through 0.2 μM filters and concentrated by passage through an Amicon concentrator with a 10 kDa cut-off membrane to collect secreted products. Secreted products from *B. malayi* microfilariae are designated *Bm*SP, whereas those from *T. spiralis* infective larvae are designated *Ts*SP. Somatic extracts were obtained by homogenisation in 25 mM Tris, pH 7.0, 0.25 mM *η*-dodecyl-β-d-maltoside, and protein concentrations determined by the BCA microplate assay (Pierce). All procedures involving animals were approved by the Imperial College Ethical Review Committee and performed under licence from the UK Home Office. Animal care and maintenance was performed by College staff as directed by the licence and institutional guidelines.

### 2.2 Chemotaxis assay

Chemotaxis assays were performed in 96-well chemotaxis chambers using membranes with a 5 μM pore diameter (Neuroprobe). Cells were isolated from venous blood of healthy donors, granulocytes isolated by standard procedures and suspended in Gated Autofluorescence/Forward Scatter (GAFS) buffer (HBSS without Mg^2+^ and Ca^2+^ supplemented with 0.1% BSA, 10 mM Hepes, pH 7.2, and 10 mM glucose). Chemotaxis plates were blocked with GAFS buffer supplemented with 1% BSA for 30 min at 37 °C in a humidified chamber prior to assay. Recombinant human C5a (Sigma) was used as the chemoattractant at a concentration of 10 nM, and pre-incubated with parasite secreted products in the presence or absence of protease inhibitors at 37 °C. Reactions were applied to the chemotaxis plate together with human granulocytes at a density of 4 × 10^6^ cells ml^−1^, and incubated for 2 h at 37 °C, 5% CO_2_. Cells were quantified by flow cytometry, with the chemotactic index representing the number of cells that migrated towards the chemoattractant divided by the number of cells that migrated to GAFS buffer alone. Phenylmethylsulphonyl fluoride (PMSF), l-transepoxysuccinyl-leucylamido-[4-guanidino]butane (E64) and pepstatin were purchased from Amersham Pharmacia Biotech AB, 1,10-phenanthroline and potato carboxypeptidase inhibitor (PCI) were from Sigma, and guanidinoethylmercaptosuccinic acid (GEMSA) was from EMB Chemicals. Statistical analysis of data in which two groups were compared was by Student’s *t*-test assuming unequal variance.

### 2.3 Carboxypeptidase assay

Carboxypeptidase activities were measured using a microplate colourimetric assay that detects the release of hippuric acid from hippuryl-conjugated substrates (Komura et al., 2002). Parasite products (10 μg) were incubated with 10 mM hippuryl-l-arginine or hippuryl-l-lysine (Sigma) in 25 mM Tris, pH 8.0, 1 mM CoCl_2_ in a total volume of 15 μl at 37 °C for 2 h. The reaction was stopped on ice, 100 μl 0.25 M phosphate buffer, pH 8.3 added, and further developed by the addition of 75 μl 3% cyanuric acid in 1,4 dioxane. After 2 min agitation, the plate was centrifuged at 3000*g* for 10 min, 100 μl of the supernatant was transferred to a fresh plate and absorbance at 405 nm determined. Carboxypeptidase activity was determined from hippuric acid standards, with porcine pancreatic carboxypeptidase B (Sigma) as a positive control. One unit of activity is equivalent to the hydrolysis of 1 μmol substrate at 37 °C per min.

### 2.4 Tricine buffered PAGE

C5a was radiolabelled with ^125^I using the chloramine T method (Hunter and Greenwood, 1962), and pre-incubated with 5 μg ml^−1^ of *Bm*SP made up to 20 μl with PBS for 2 h at 37 °C in the presence or absence of protease inhibitors. The reaction was stopped by the addition of SDS loading buffer, and the samples resolved on 18% SDS–polyacrylamide tricine-buffered gels (Schagger, 2006) which were fixed, dried and exposed to autoradiography.

## 3 Results

### 3.1 *Bm*SP inactivate C5a-mediated chemotaxis of human granulocytes

**Fig. 1:**
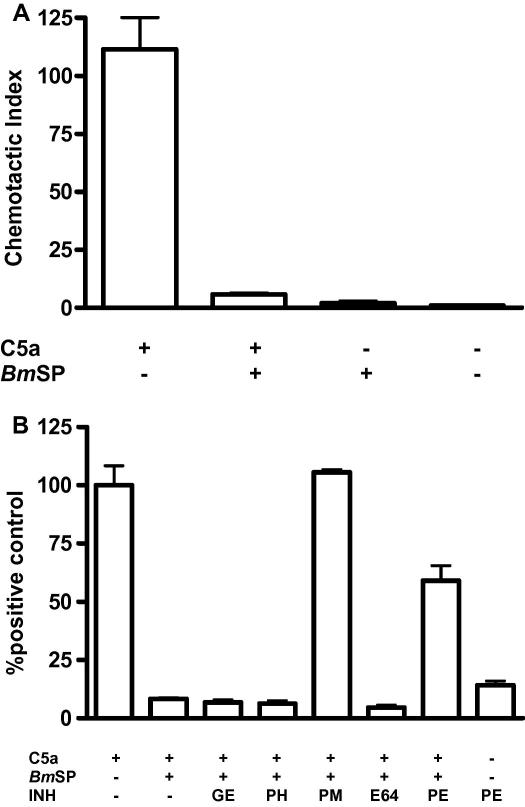
*Brugia malayi* secreted products inhibit anaphylatoxin C5a-mediated chemotaxis. (A) *Brugia malayi* microfilarial secreted products (*Bm*SP) at a concentration of 5 μg ml^−1^ were pre-incubated with 10 nM C5a for 1 h at 37 °C, and reactions were used as the chemoattractant in a human granulocyte chemotaxis assay. Data show the means + SD for results obtained in triplicate, *n *= 2. (B) The following panel of inhibitors (INH) were added to 5 μg ml^−1^*Bm*SP before pre-incubation with 10 nM C5a for 1 h at 37 °C: 1 mM guanidinoethylmercaptosuccinic acid (GE), 100 μM 1,10 phenanthroline (PH), 1 mM phenylmethylsulphonyl fluoride (PM), 2 μM E64 and 1 μM pepstatin (PE). Data are expressed as the percentage of the positive control, and represent the means + SD for results obtained in triplicate, *n *= 2.

We initially set out to examine whether *Bm*SP had any effect on complement-mediated attraction of granulocytes. Pre-incubation of C5a with 5 μg ml^−1^ *Bm*SP for 30 min at 37 °C almost completely abolished chemotaxis of human granulocytes (95% reduction with respect to control values), whereas incubation of granulocytes with *Bm*SP alone had minimal chemotactic effect (Fig. 1A). Using a panel of protease inhibitors, it was observed that the reduction in chemotaxis could be completely blocked by the serine protease inhibitor PMSF (Fig. 1B). Pepstatin also partially blocked the action of *Bm*SP, but induced significant chemotaxis when presented to granulocytes alone, the only inhibitor tested which displayed this effect (Fig. 1B). GEMSA, 1,10-phenanthroline or E64 did not inhibit the action of *Bm*SP, suggesting that carboxypeptidases, metalloproteases or cysteine proteases, respectively, were not involved in the inhibitory effect.

### 3.2 Microfilarial secreted products cleave C5a

**Fig. 2:**
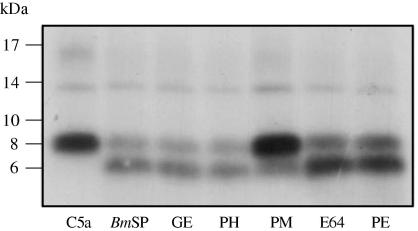
Cleavage of anaphylatoxin C5a by *Brugia malayi* secreted products (*Bm*SP). Iodinated C5a was pre-incubated with 5 μg ml^−1^*Bm*SP for 2 h at 37 °C before being separated on an 18% SDS–polyacrylamide gel using a tricine based running buffer. The protease inhibitors guanidinoethylmercaptosuccinic acid (GE), 1,10 phenanthroline (PH), phenylmethylsulphonyl fluoride (PM), l-transepoxysuccinyl-leucylamido-[4-guanidino]butane (E64) and pepstatin (PE) were added to the pre-incubation at the concentrations stated in Fig. 1B. The gel was dried and the bands detected by autoradiography. Molecular weight markers are shown in kDa. This experiment was performed twice with identical results.

In order to confirm that C5a was being proteolytically cleaved, iodinated C5a was incubated with *Bm*SP and separated by tricine buffered SDS–PAGE to resolve small peptide fragments. C5a, with a mass of 8 kDa, was cleaved into a fragment approximately 6 kDa in size (Fig. 2). As with the chemotaxis assay, PMSF inhibited cleavage, whereas GEMSA, 1,10 phenanthroline, E64 and pepstatin did not, suggesting that a serine protease was responsible.

### 3.3 *Ts*SP also inactivate C5a

**Fig. 3:**
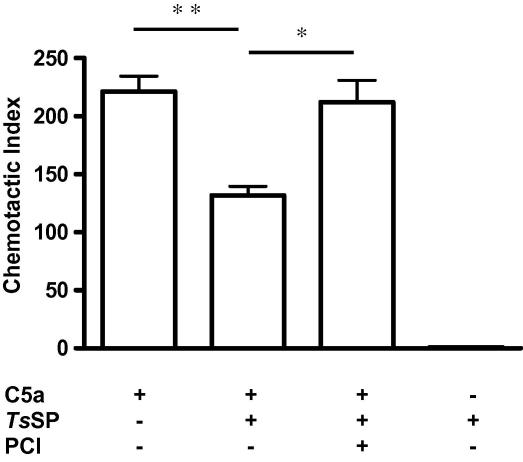
The anaphylatoxin C5a pre-incubated with *Trichinella spiralis* secreted products shows reduced chemotactic potential towards human granulocytes. *Trichinella spiralis* secreted products (*Ts*SP) at a concentration of 50 μg ml^−1^ were pre-incubated with 10 nM C5a for 60 min at 37 °C. Potato carboxypeptidase inhibitor (PCI) was added to the pre-incubation at a final concentration of 1 mM. Data show mean values + SD for results obtained in triplicate, *n *= 3. Significant differences between groups are indicated as ^∗^*P *< 0.05 and ^∗∗^*P *< 0.01.

In order to determine whether tissue-invasive larvae of another parasitic nematode could also affect complement-mediated chemotaxis, we tested *Ts*SP. Initial titrations indicated that higher quantities of *Ts*SP were required for significant effects. Thus, when C5a was incubated with 50 μg ml^−1^ *Ts*SP for 30 min at 37 °C, there was a 43% reduction in the number of human peripheral blood granulocytes which migrated towards the chemoattractant. Again, no significant chemotaxis was observed with parasite secreted products alone (Fig. 3). Unlike the effect with *Bm*SP, 1 mM PCI completely blocked the inhibition of chemotaxis shown by *Ts*SP (Fig. 3), suggesting that inactivation of C5a was effected by a carboxypeptidase.

### 3.4 Both adult and larval stages of *T. spiralis* and *B. malayi* secrete carboxypeptidases

**Fig. 4:**
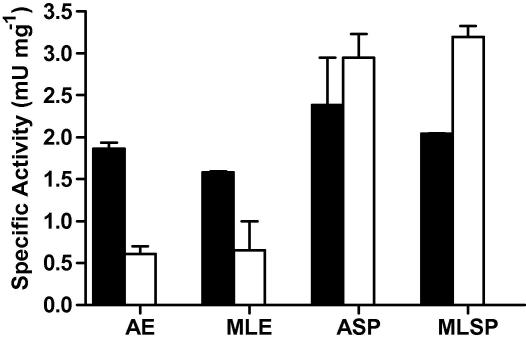
Detection of carboxypeptidase activities in *Trichinella spiralis* samples. Somatic extracts of *T. spiralis* adult (AE) and muscle stage larvae (MLE) together with the secreted products of adult (ASP) and muscle stage larvae (MLSP) parasites were tested for the presence of carboxypeptidase activity using hippuryl-l-lysine (■) or hippuryl-l-arginine (□) synthetic substrates. The samples (10 μg) were incubated with 15 mM substrate for 2 h at 37 °C. Data show the means + SE for results obtained in triplicate, *n *= 3.

**Fig. 5:**
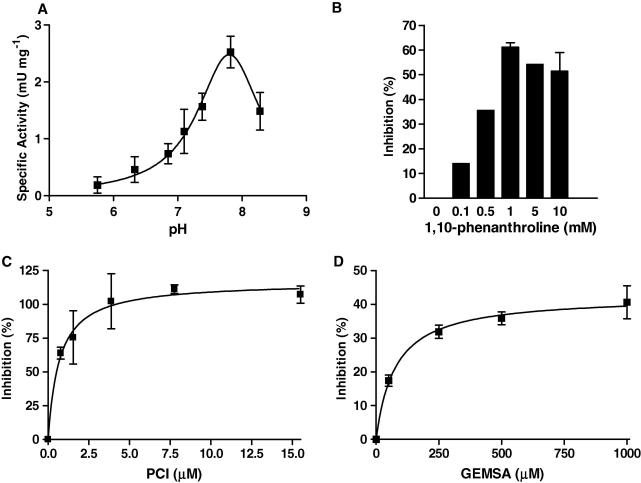
Characteristics of the carboxypeptidase activity in *Trichinella spiralis* muscle stage larvae secreted products. (A) pH dependency was determined in Tris–maleate buffer using a pH range of 5.6–8.3. (B) Inhibition of carboxypeptidase activity by the addition of 1,10-phenanthroline. (C and D) Inhibition by the zinc metallocarboxypeptidase specific inhibitors, potato carboxypeptidase inhibitor (PCI) and guanidinoethylmercaptosuccinic acid (GEMSA). Data are expressed as the percentage inhibition from the positive control. Values represent the means ± SE for results obtained in triplicate, *n *= 3. All experiments used hippuryl-l-arginine as the substrate.

A colourimetic assay with hippuryl-l-lysine and hippuryl-l-arginine as substrates was employed to detect carboxypeptidase activities in different stages and preparations of both parasite species, as this assay was originally utilised to identify serum carboxypeptidases which inactivate complement-derived anaphylatoxins (Bokisch and Muller-Eberhard, 1970). Activity against both substrates was detected in somatic extracts of *T. spiralis* infective larvae and adults. Initial optimisation indicated that maximal activity was observed with the inclusion of 1 mM CoCl_2_, and thus this was included in the standard assay buffer. Somatic extracts are likely to contain multiple carboxypeptidase activities. However, it was notable that whilst these preparations preferentially cleaved hippuryl-l-lysine, secreted products were enriched in activity against the hippuryl-l-arginine substrate (Fig. 4). The carboxypeptidase activity in infective larval secreted products was characterised further. It had a pH optimum of 7.9 (Fig. 5A), and like many metallocarboxypeptidases was inhibited by 1,10 phenanthroline (Fig. 5B). Fig. 5C and D shows that the carboxypeptidase was only partially sensitive to inhibition with GEMSA, whereas PCI was more strongly inhibitory.

**Fig. 6:**
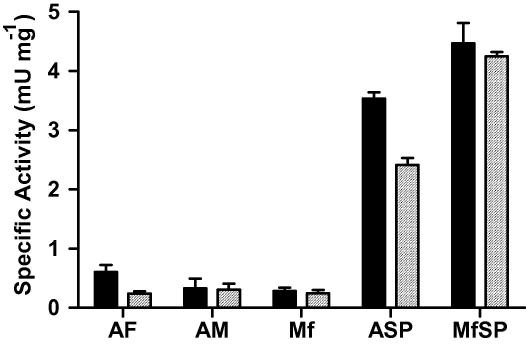
Carboxypeptidase activities in different life stages of *Brugia malayi*. *Brugia malayi* somatic extracts from adult female (AF), adult male (AM) and microfilarial (Mf) stages as well as secreted products from adults (ASP) and microfilaria (MfSP) were tested for the presence of carboxypeptidase activity using hippuryl-l-lysine (■) or hippuryl-l-arginine (□) synthetic substrates. The samples (10 μg) were incubated with 15 mM substrate for 2 h at 37 °C. Data shown are the means + SE for results obtained in triplicate, *n *= 2.

Somatic extracts of *B. malayi* adults and microfilariae had relatively low carboxypeptidase activities, but these were again substantially enriched in secreted products, and those of microfilariae had particularly high levels of activity against both lysine and arginine substrates (Fig. 6). The limiting material secreted in vitro by these parasites precluded any detailed characterisation, although 1 mM GEMSA was observed to completely block activity (data not shown).

## 4 Discussion

The anaphylatoxin C5a is a potent chemoattractant for myeloid cells, particularly neutrophils, which express high levels of the C5a receptor. In addition to chemotaxis, C5a has multiple effects on neutrophils, for example upregulation of adhesion molecules, assembly and activation of the NADPH oxidase, degranulation and mediator release (Webster et al., 1980; Kishimoto et al., 1989; Ehrengruber et al., 1994). Other effects of C5a which promote inflammation include recruitment and degranulation of mast cells and basophils (el-Lati et al., 1994; Nilsson et al., 1996), vasodilation and contraction of smooth muscle (Ward, 2004).

The activities of C5a and the other complement-derived anaphylatoxins are normally regulated by zinc metallocarboxypeptidases in the serum which remove the C-terminal arginine residue, resulting in inactivated or *des-Arg* forms. Whilst C3a and C4a are completely inactivated by removal of the C-terminal arginine, C5a retains approximately 10% of its chemotactic activity, which may explain the partial inactivation of chemotaxis effected here by *Ts*SP.

Carboxypeptidase R (CPR) exists as an inactive zymogen in plasma, bound to plasminogen and activated by plasmin or thrombin (Wang et al., 1994; Sato et al., 2000). CPR is an acute phase protein with equal activity against arginine and lysine substrates, but it preferentially hydrolyses C5a over C3a (Campbell et al., 2002). In addition to inactivation of anaphylatoxins, CPR has an important role in the regulation of coagulation and fibrinolysis (Bajzar et al., 1995). In contrast, carboxypeptidase N (CPN) is present in the active state (Levin et al., 1982) and its expression is not influenced by inflammation. It shows a preference for lysine substrates and preferentially hydrolyses C3a over C5a (Campbell et al., 2002). With no clear role in coagulation, CPN may serve as a constitutive inactivator of tickover-generated C3a.

In this study, C5a was inactivated by a serine protease from *B. malayi* and a carboxypeptidase from *T. spiralis*. Carboxypeptidases were secreted by adult and larval stages of both parasites but sufficient quantities of secreted proteins could only be recovered from *T. spiralis* cultures for characterisation. Mammalian plasma CPR is inhibited preferentially by PCI over GEMSA and is partially inhibited by the addition of cobalt. In contrast, CPN is activated by the addition of cobalt, inhibited by GEMSA but relatively insensitive to PCI (Levin et al., 1982; Hendriks et al., 1990; Wang et al., 1994). The *T. spiralis* carboxypeptidase activity was thus most similar to CPR in inhibitor sensitivities, but was slightly activated by the addition of cobalt, as was the *B. malayi* secreted carboxypeptidase. It is possible that parasite secreted products contain several carboxypeptidases, which would confound more detailed analysis.

It was interesting to note that secreted products from both *B. malayi* microfilariae and *T. spiralis* infective larvae were not directly chemotactic to granulocytes, at least in vitro. In vivo studies on patients infected with *Onchocerca volvulus* also suggest that the parasites per se do not release chemotactic signals, but that neutrophil accumulation around adult worms/nodules is driven by *Wolbachia* endobacteria. This was observed in vivo by treatment of patients with doxycycline, and in vitro by parasite extracts from worms removed from untreated or treated patients. Thus, extracts of parasites surgically removed from untreated patients showed neutrophil chemotactic activity and induced TNF-α and IL-8 production by monocytes in vitro, in contrast to extracts of parasites removed from patients obtained after doxycycline treatment (Brattig et al., 2001).

Recent studies have shown that complement plays a role in granulocyte recruitment and parasite attrition early in primary infection (i.e. in the absence of antibody) of mice with *N. brasiliensis* (Giacomin et al., 2008b) and contributes to killing of *Strongyloides stercoralis* larvae (Kerepesi et al., 2006). This raises the question of how complement is activated during nematode infection prior to antibody production. Giacomin et al. (2005) utilised sera from mice deficient in specific complement components to examine activation and deposition on the surface of different stages of *N. brasiliensis*. They observed substantial deposition of C3 on the surface of infective L3s, which occured as a result of alternative activation, and that this was accompanied by a high level of leucocyte adherence. Interestingly, C3 deposition was almost completely abolished on L4s recovered 24 h later from the lungs, but thereafter, L4 and adult worms progressively reacquired the ability to fix C3 on their surface, with the lectin pathway playing a role for the latter. These data suggest that *N. brasiliensis* infective larvae promote complement fixation and are susceptible to leucocyte-mediated attack immediately after invasion of the definitive host, but rapidly avoid this arm of the immune response whilst still migrating through somatic tissues. Similar loss of complement fixation and/or cellular adherence is displayed by *O. volvulus* and *Dirofilaria immitis* as they make the transition from L3 to L4 stages, and this may represent an evasion mechanism to promote establishment (Abraham et al., 1988; Brattig et al., 1991). *Onchocerca volvulus* microfilariae utilise another method of inactivating complement by binding factor H which, in the presence of factor I, promotes the cleavage of C3b to iC3b, and restricts amplification of the alternative pathway (Meri et al., 2002).

The lectin pathway has also been shown to be responsible for activation of complement on the surface of *T. spiralis* and *B. malayi*. Mannose-binding lectin (MBL) binds oligosaccharides on the surface of *T. spiralis* muscle stage larvae, and binds to glycoproteins in their secreted products (Gruden-Movsesijan et al., 2003). Murine MBL-A binds the surface of *B. malayi* microfilariae with subsequent activation of C3 (Carter et al., 2007). Intriguingly, microfilariae were observed to survive for longer in MBL-A^−/−^ deficient mice than in matched wild-type animals, and this was accompanied by a profound defect in antigen-specific IgM production (Carter et al., 2007). This could explain increased parasite survival in these animals, as IgM has been shown to be important for clearance of *B. malayi* microfilariae (Thompson et al., 1981; Gray and Lawrence, 2002). It was suggested that the role of MBL underlying effective anti-microfilarial immune responses may be to complex parasite glycosylated antigens for uptake by antigen presenting cells (Carter et al., 2007) and, interestingly, polymorphisms in the promoter region in MBL2 have been observed to be associated with susceptibility to human lymphatic filariasis (Choi et al., 2001).

Although *N. brasiliensis* L3s are trapped in the skin of IL-5 transgenic mice for extended periods, indicative of susceptibility to eosinophil-mediated attack (Daly et al., 1999), other nematodes such as *T. spiralis* and *Toxocara canis* appear unaffected (Sugane et al., 1996; Hokibara et al., 1997; Dent et al., 1999), and eosinophilia may actually impair host resistance to *T. spiralis* (Dent et al., 1997). More recent work suggests that eosinophils promote survival of *T. spiralis* by skewing cytokine production and thereby inhibiting nitric oxide synthesis (Fabre et al., 2009). Secreted products from *T. canis* infective larvae inhibit the deposition of C3 and adherence of eosinophils to *N. brasiliensis*, suggesting that a proteolytic activity may degrade C3 (Giacomin et al., 2008a). Moreover, addition of *T. canis* secreted products to the inoculum of *N. brasiliensis* L3s enhances the number of parasites which migrate to the lungs of IL-5 transgenic mice, indicating that the protective effect is also operative in vivo (Giacomin et al., 2008a).

Enzymes secreted by nematodes have been shown to degrade other pro-inflammatory chemotactic factors. For example, eotaxin is cleaved and inactivated by a secreted metalloprotease from *Necator americanus* (Culley et al., 2000), and it has been observed that *T. spiralis* and *N. brasiliensis* secrete metallo- and serine proteases which cleave eotaxin (Weller, 2002). A problem with these observations and the current study is determining which are natural and which are adventitious substrates for any given proteolytic enzyme. For example, carboxypeptidases secreted by parasitic nematodes may have other targets, and indeed the mammalian serum CPR plays an additional role in the regulation of coagulation and fibrinolysis (Bajzar et al., 1995). The most satisfactory means of attempting to answer this question would be via gene deletion or functional knockout of the enzyme and then assessing the effect on parasite infection and the ensuing inflammatory response. Although RNA interference appears to hold some promise, there is as yet no robust means to apply this to animal parasitic nematodes (Geldhof et al., 2007). Hopefully this situation will be resolved in the near future so that we can more rigorously analyse the role of putative immunomodulatory or virulence factors.
